# Evaluation of HuoXueHuaYu therapy for nonalcoholic fatty liver disease: a systematic review and meta-analysis of randomized controlled trial

**DOI:** 10.1186/s12906-019-2596-3

**Published:** 2019-07-19

**Authors:** Yunfei Cai, Qiuer Liang, Weihao Chen, Minghao Chen, Ruixue Chen, Yun Zhang, Ya Xiao, Liguo Chen

**Affiliations:** 1 School of Traditional Chinese Medicine, Jinan University, Guangzhou, China; 2 Division of Histology and Embryology, Key Laboratory for Regenerative Medicine of the Ministry of Education, Medical College, Jinan University, Guangzhou, China; 3 International Joint Laboratory for Embryonic Development and Prenatal Medicine, Medical College, Jinan University, Guangzhou, China; 4 Second Affiliated Hospital, Guangzhou Medical University, Guangzhou, China

**Keywords:** Huoxuehuayu, NAFLD, Meta-analysis

## Abstract

**Background:**

To evaluate the effectiveness and safety of HuoXueHuaYu (HXHY) therapy in treating nonalcoholic fatty liver disease (NAFLD) through a systematic review and meta-analysis.

**Methods:**

We performed comprehensive searches on Embase, Pubmed, Cochrane Library, CNKI, VIP and Wanfang databases up to June 2017 for randomized controlled trials using HXHY in the treatment of NAFLD compared with conventional treatment.

**Results:**

This meta-analysis included 13 studies involving 1429 patients which 775 patients belonged to HXHY group and 654 patients belonged to conventional treatment group. The results of meta-analysis showed that HXHY can significantly improve B ultrasonic level (OR = 2.33; 95% CI:1.60, 3.40; *P* < 0.00001) of NAFLD compared with conventional treatment. As to lipids, HXHY was tested to be better on reduction of total cholesterol (TC) (MD = -0.38, 95% CI: − 0.48, − 0.29; *P* < 0.00001) and triglyceride (TG) (MD = -0.31; 95% CI: − 0.37, − 0.24; *P* < 0.00001) than conventional treatment. HXHY also had a greater beneficial effect on liver function in reducing alanine transaminase (ALT) (MD = -1.69; 95% CI: − 2.24, − 1.14; *P* < 0.00001) and aspartate transaminase (AST) (MD = -22.53; 95% CI: − 33.16, − 11.90; *P* < 0.00001) compared with conventional treatment. HXHY can also significantly improve the effective rate (OR = 3.55; 95% CI:2.65, 4.76; *P* < 0.00001) compared with conventional treatment. No serious adverse reactions were reported.

**Conclusions:**

HXHY seems to be an effective and safe therapy for NAFLD. It is suggested that further study of HXHY in the treatment of NAFLD requires trials with rigorous design, multicenter, large-scale and high-quality worldwide.

## Background

Nonalcoholic fatty liver disease (NAFLD) is a common chronic liver disease, with prevalence between 14 and 45% in the world [1, 2]. One clinical study suggested that about 1/3 of NAFLD patients could develop into nonalcoholic steatohepatitis (NASH), and once they developed into NASH, the risks of liver cirrhosis, liver cancer, and liver failure might increase significantly [3].

The main therapies of NAFLD in conventional treatment are lifestyle intervention and drug therapy. Lifestyle intervention is hard to be applied due to lack of compliance. Therefore, drug therapy plays an important role in treating patients with NAFLD. Vitamin E and pioglitazone showed positive effects on liver function and lipid deposition. However, in spite of some beneficial effects, vitamin E does not have therapeutic effect on liver fibrosis and pioglitazone causes weight gain [4]. Other drugs such as metformin, orlistat and statins were of limited benefit [5]. Therefore, development of an effective therapy is of significant importance for NAFLD.

In Traditional Chinese medicine (TCM), chronic liver diseases are usually considered to be accompanied by blood stasis [6, 7]. Promoting blood circulation (Chinese name in pinyin “Huo Xue HuaYu” (HXHY)) is an important therapy in the treatment of NAFLD [8]. A previous study compared different TCM therapies for NAFLD and indicated that HXHY therapy was superior to other therapies in treating patients with NAFLD [9]. More and more traditional Chinese herbs with the function of activating blood circulation have been proved to be effective in treating NAFLD [10]. Though there are several clinical trials suggested that HXHY therapy has therapeutic potential in treating NAFLD, the effectiveness of HXHY has not been assessed in system. Therefore, the present meta-analysis aimed to evaluate the effectiveness and safety of HXHY in treating NAFLD by a systematic review and meta-analysis of randomized controlled trial (RCTs) to provide evidence for clinical practice.

## Methods

### Search strategy

The study was performed following the PRISMA guidelines [11]. The literature search was conducted using Cochrane Library (1993 to June 2017), the PubMed database (2000 to June 2017), the Embase database (1974 to June 2017), the China National Knowledge Infrastructure database (1979 to June 2017), the Wanfang database (1982 to June 2017), the VIP database (1989 to June 2017). Search terms were (NAFLD OR nonalcoholic fatty liver disease OR fatty liver disease) AND (HuoXueHuaYu OR activating blood circulation OR Chinese medicine OR herbs OR herbal medicine).

### Study selection

Inclusion criteria were as following: (a) Patients were diagnosed with NAFLD; (b) The trial was claimed to be a RCT; (c) The formula used in the study included HXHY-class herbs. The herbs which have the function of activating blood circulation were defined as HXHY-class herbs. The most commonly used HXHY-class herbs in clinical practice are *Salvia miltiorrhiza* (Dan shen), *Ligusticum wallichii* (Chuan xiong), *Hawthorn* (Shan zha), *Rhizoma curcumae longae* (Jiang huang), *Curcuma aromatic* (Yu jin), *Panax pseudo-ginseng* (Tian qi), *Peach kernel* (Tao ren), *Rhizoma sparganii* (San leng), *Curcuma zedoaria* (E zhu), *Carthami Flos* (Hong hua), *Eupatorium japonicum* (Ze lan), *Corydalis Rhizoma* (Yan hu suo), *Semen vaccariae* (Wang bu. liu xing), etc.; (d) The study compared the efficacy of HXHY with conventional treatment.

Exclusion criteria were as following: (a) duplicated or redundant study; (b) nonhuman studies; (c) nonrandomized controlled trials.

### Outcome indicators

The primary outcome was the level of type-B ultrasonic of liver, and the secondary outcomes were levels of total cholesterol (TC), triglyceride (TG), alanine transaminase (ALT) and aspartate transaminase (AST) and the effective rate.

### Data extraction and analysis

Data Extraction of the included studies were performed by two researchers independently. They discussed and recorded any disagreement. The third researcher resolved the disagreement that could not be resolved through discussion. Cochrane Risk of Bias Tool was used to evaluate the quality of RCTs.

Mean difference (MD) was reported for TC, TG, AST and ALT. Odds ratio (OR) was reported for B ultrasonic level and effective rate using Review Manager software (RevMan 5.3). 95% confidence interval (CI) will be used as an effective size for the combined analysis. *I*^2^ statistics is used to estimate heterogeneities. If there is no heterogeneity (*I*^*2*^ < 50% and *P* > 0.1), a fixed-effect model is used to synthesize the data; Otherwise, if there is heterogeneity (50% < *I*^*2*^ < 75%), a random-effect model was applied. When *I*^2^ > 75%, subgroup analysis or sensitivity analysis was performed to identify the causes of the heterogeneity. A funnel plot was selected to assess the publication bias.

## Results

### Study selection and quality evaluation

**Fig. 1:**
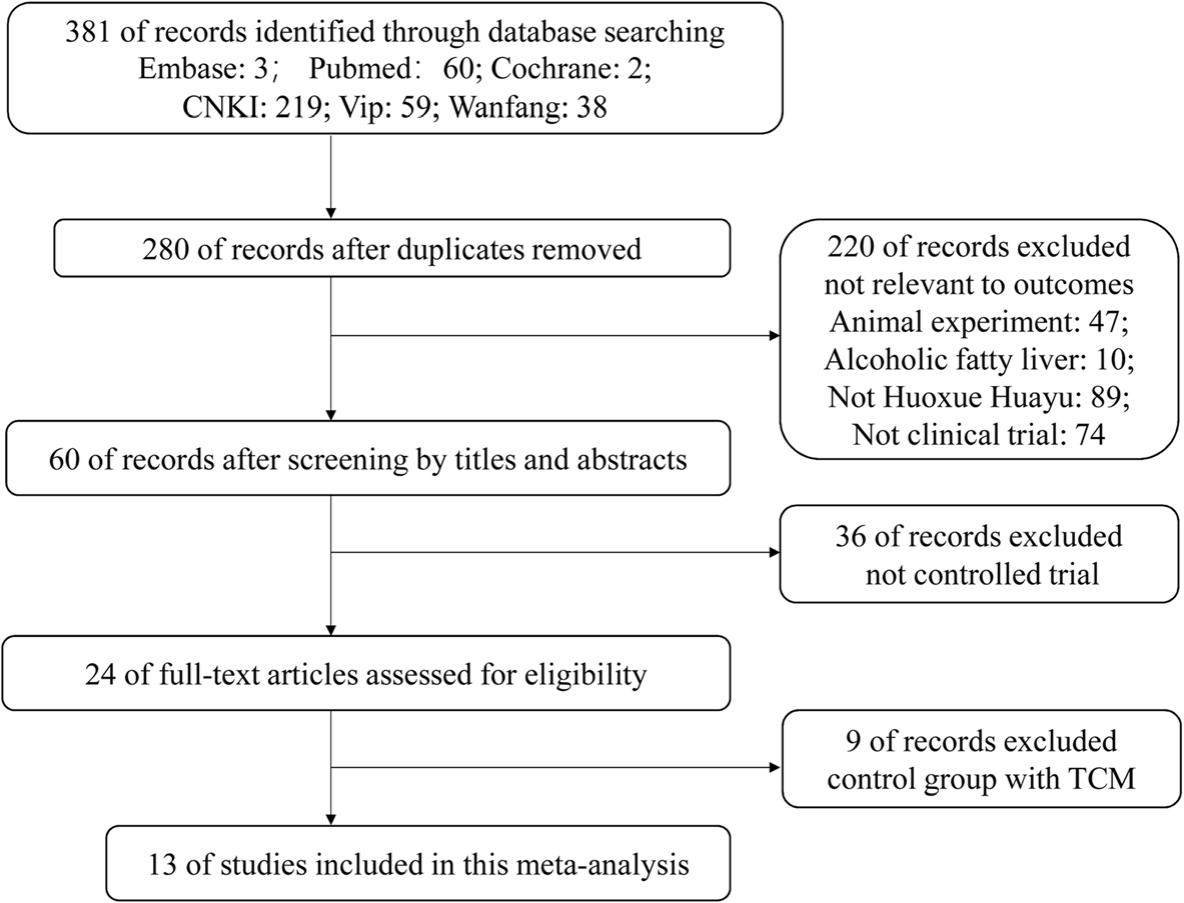
Flowchart of study selection

Firstly, we searched out 381 studies completely and then keep 280 studies after deleting repeated records. Eliminating case reports, reviews and animal researches, we achieved 13 studies [12–24] (Fig. 1). The 13 studies included 1429 cases in total. Of which, 775 belonged to the HXHY therapy group and 654 belonged to the conventional treatment group. The patients included in each study were all classified as NASH. Table 1 listed the characteristics of the studies. Table 2 listed the compositions of the herbal formulae.

**Table 1:** Characteristics of the 13 included studies

Author(s), year	NAFLD diagnostic criteria	NAFLD classification	Patients included	Men (%)	Age (years)	Interventions		Treatment duration
						Experimental	Control	
Wan Jun 2014 [12]	Chinese guidelines of NAFLD for 2010	NASH	60	NA	NA	Danhebaohe decoction	Simvastatin	12 weeks
Hairong Liu 2010 [13]	Chinese diagnostic criteria of NAFLD for 2003	NASH	190	46.8	47.9	Jianglanzhigan decoction	Fluvastatin	4 weeks
Guangjun Tian 2009 [14]	Chinese guidelines of NAFLD for 2006	NASH	80	32.5	36.4	Tiaoganxiaozhi decoction	Essentiale	24 weeks
Huiwu Zhu 2008 [15]	Chinese guidelines of NAFLD for 2006	NASH	100	27.0	45.3	Shuganhuoxue decoction	Inositol nicotinate and VitB	8 weeks
Xiangfa Zou 2008 [16]	Chinese diagnostic criteria of NAFLD for 2003	NASH	60	38.3	45.4	Qinggan decoction	Simvastatin	4 weeks
Qikai Wu 2006 [17]	Chinese diagnostic criteria of NAFLD for 2003	NASH	74	NA	NA	Shanzhabeimu decoction	Ethyl Polyenoate Soft Capsules	12 weeks
Guo Yan 2006 [18]	Chinese diagnostic criteria of NAFLD for 2003	NASH	400	33.0	50.8	Shuganliqihuoxue san	VitB_1_,VitB_2_,VitB_6_ and polysaccharide sulphate	8 weeks
Li Jin 2006 [19]	Chinese diagnostic criteria of NAFLD for 2003	NASH	80	23.8	43.1	Huoxuehuayu decoction	Compound Methionine and Choline Bitartrate Tablets	12 weeks
Suicheng Guo 2006 [20]	Chinese diagnostic criteria of NAFLD for 2003	NASH	130	42.3	39.0	Qinglengezhu decoction	Ethyl Polyenoate Soft Capsules	4 weeks
Huaien Xu 2005 [21]	Chinese diagnostic criteria of NAFLD for 2003	NASH	67	22.4	NA	Jiangzhiyigan decoction	Inositol, fenofibrate, VitC and Compound choline tablets	16 weeks
Minfang Zhang 2002 [22]	Chinese diagnostic criteria of NAFLD (draft)	NASH	58	37.9	51.0	Quzhi decoction	VitC,VitE and polysaccharide sulphate	6 weeks
Lu Xia 2001 [23]	Chinese diagnostic criteria of NAFLD (draft)	NASH	66	48.5	39.0	Huoxue Huayu decoction	VitC, Choline and Inositol	6 weeks
Xiaoming Zhang 1997 [24]	Chinese diagnostic criteria of NAFLD (draft)	NASH	64	34.4	43.0	JianpihuazhuHuoxueHuayu decoction	VitB_1_, VitC and polysaccharide sulphate	7 weeks

*NA* Not available, *NASH* Nonalcoholic steatohepatitis

**Table 2:** The ingredients of each formula

Author(s), year	Ingredients of each formula				
Wan Jun 2014	Hawthorn (Shan zha)	Salvia miltiorrhiza (Dan shen)	Panax pseudo-ginseng (Tian qi)	Lotus leaf (He ye)	Pinellia ternate (Ban xia)
	Poria cocos (Fu ling)	Citri reticulatae (Chen pi)	Fructus aurantii (Zhi qiao)	*Forsythia suspensa* (Lian qiao)	Albiziae Cortex (He huan pi)
	Medicated leaven (Shen qu)	Semen raphanin (Lai fu zi)	Malt (Mai ya)	Glycyrrhizae preparata (Zhi gan cao)	*Sedum sarmentosum* Bunge (Chui pen cao)
Hairong Liu 2010	Salvia miltiorrhiza (Dan shen)	Ligusticum wallichii (Chuan xiong)	Rhizoma curcumae longae (Jiang huang)	Curcuma aromatic (Yu jin)	Gynostemma pentaphyllum (Jiao gu lan)
	Bupleurum (Chai hu)	Poria cocos (Fu ling)	Scutellaria baicalensis (Huang qin)	*Acorus calamus* (Shi chang pu)	Magnolia officinalis (Hou pu)
	*Gardenia jasminoides* (Zhi zi)	Artemisia capillary (Yin chen)			
Guangjun Tian 2009	Salvia miltiorrhiza (Dan shen)	Hawthorn (Shan zha)	Curcuma aromatic (Yu jin)	Bupleurum (Chai hu)	Poria cocos (Fu ling)
	Fructus aurantii (Zhi qiao)	Citri reticulatae (Chen pi)	Pinellia ternate (Ban xia)	*Polygonum multiflorum* (He shou wu)	Cassia Seed (Jue ming zi)
	Alisma orientale (Ze xie)	Endothelium corneum gigeriae galli (Ji nei jin)			
Huiwu Zhu2008	Salvia miltiorrhiza (Dan shen)	Hawthorn (Shan zha)	Curcuma aromatic (Yu jin)	Ligusticum wallichii (Chuan xiong)	Citri reticulatae (Chen pi)
	Pinellia ternate (Ban xia)	Alisma orientale (Ze xie)	Cassia Seed (Jue ming zi)	Polygonum multiflorum (He shou wu)	Bupleurum (Chai hu)
	Radix paeoniae rubra (Chi shao)				
Xiangfa Zou2008	Salvia miltiorrhiza (Dan shen)	Hawthorn (Shan zha)	Bupleurum (Chai hu)	Cassia Seed (Jue ming zi)	Alisma orientale (Ze xie)
	Artemisia capillary (Yin chen)	Lycium chinensis (Gou qi)	*Rheum officinale* (Da huang)	Glycyrrhizae preparata (Zhi gan cao)	
Qikai Wu2006	Hawthorn (Shan zha)	Fritillaria thunbergii (Bei mu)	Alisma orientale (Ze xie)	Artemisia capillary (Yin chen)	Trichosanthes kirilowii Maxim (Gua lou)
	*Polygonum cuspidatum* (Hu zhang)				
Guo Yan 2006	Salvia miltiorrhiza (Dan shen)	Peach kernel (Tao ren)	Bupleurum (Chai hu)	Radix paeoniae rubra (Chi shao)	Angelica sinensis (Dang gui)
	Endothelium corneum gigeriae galli (Ji nei jin)	Radix Aucklandiae (Mu Xiang)	Cyperi rhizome (Xiang fu)	*Paeonia lactiflora* Pall (Bai shao)	Glycyrrhizae preparata (Zhi gan cao)
Li Jin2006	Salvia miltiorrhiza (Dan shen)	Hawthorn (Shan zha)	Alisma orientale (Ze xie)	Sargassum pallidum (Hai zao)	
Suicheng Guo2006	Hawthorn (Shan zha)	Peach kernel (Tao ren)	Rhizoma sparganii (San leng)	*Curcuma zedoaria* (E zhu)	Lotus leaf (He ye)
	Radix Aucklandiae (Mu Xiang)	Angelica sinensis (Dang gui)	Radix paeoniae rubra (Chi shao)	Rheum officinale (Da huang)	Green Tangerine Peel (Qing pi)
	Carapax Amydae (Bie jia)	Fructus aurantii (Zhi qiao)	Atractylodes Lancea (Cang zhu)		
Huaien Xu2005	Salvia miltiorrhiza (Dan shen)	Hawthorn (Shan zha)	Alisma orientale (Ze xie)	Polygonum multiflorum (He shou wu)	Cassia Seed (Jue ming zi)
	Rhizoma polygonate (Huang jing)	Lotus leaf (He ye)	Polygonum cuspidatum (Hu zhang)		
MinfangZhang2002	Salvia miltiorrhiza (Dan shen)	Hawthorn (Shan zha)	Curcuma aromatic (Yu jin)	Polygonum multiflorum (He shou wu)	Cassia Seed (Jue ming zi)
	Alisma orientale (Ze xie)	Atractylodes Lancea (Cang zhu)	*Platycodon grandiflorum* (Jie geng)		
Lu Xia2001	Salvia miltiorrhiza (Dan shen)	Eupatorium japonicum (Ze lan)	Curcuma aromatic (Yu jin)	Hawthorn (Shan zha)	Corydalis Rhizoma (Yan hu suo)
	Semen vaccariae. (Wang bu. liu xing)	Radix paeoniae rubra (Chi shao)			
XiaomingZhang1997	Salvia miltiorrhiza (Dan shen)	Hawthorn (Shan zha)	Carthami Flos (Hong hua)	Atractylodes Lancea (Cang zhu)	Citri reticulatae (Chen pi)
	Poria cocos (Fu ling)	Fructus aurantii (Zhi qiao)	Paeonia lactiflora Pall (Bai shao)	Alisma orientale (Ze xie)	Polygonum multiflorum (He shou wu)
	Bupleurum (Chai hu)	*Codonopsis pilosula* (Dang shen)	Agastache rugosa (Huo xiang)	Bamboo Shavings (Zhu ru)	*Perilla frutescens* (Zi su)

**Fig. 2:**
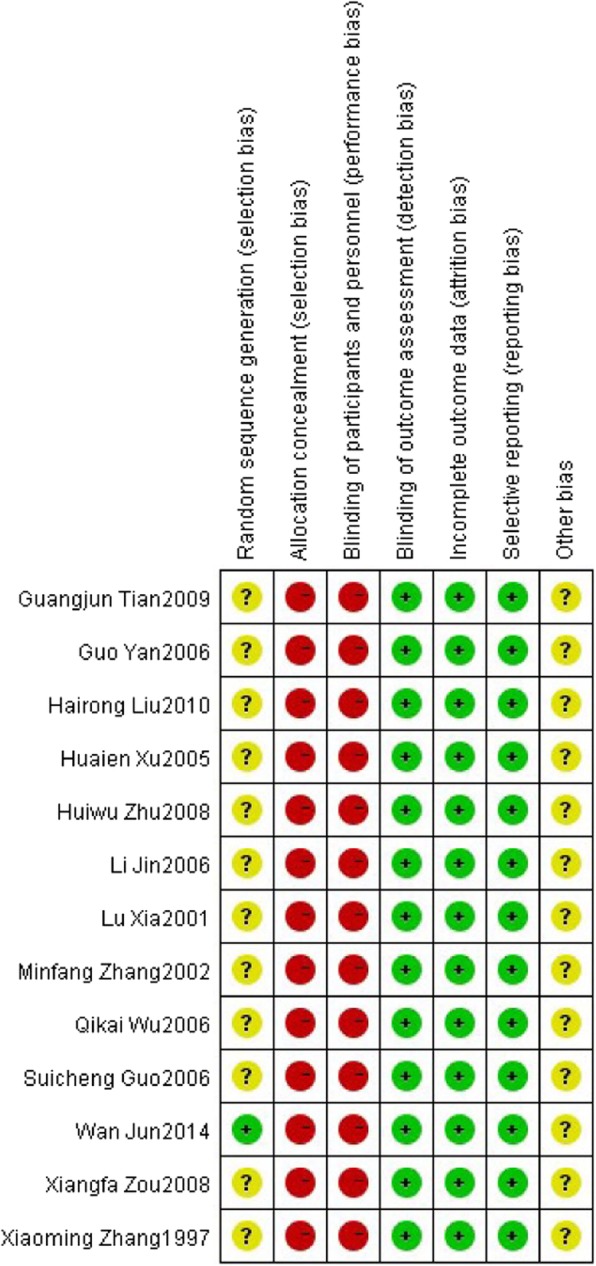
Potential risk of bias of each included study

Figure 2 showed the quality evaluation of the included studies. In terms of random sequence generation, only 1 study used a table of random number [12], while 12 studies mentioned “random”, but there was no detail of the randomization method. In the aspect of blinding, no studies mentioned blinding of the patients and personnel. In addition, no study reported allocation concealment. Selective reporting, incomplete outcome data and blinding of outcome assessment were evaluated as low risk of bias.

### The effect of HuoXueHuaYu therapy on B ultrasonic level in patients with NAFLD

**Fig. 3:**
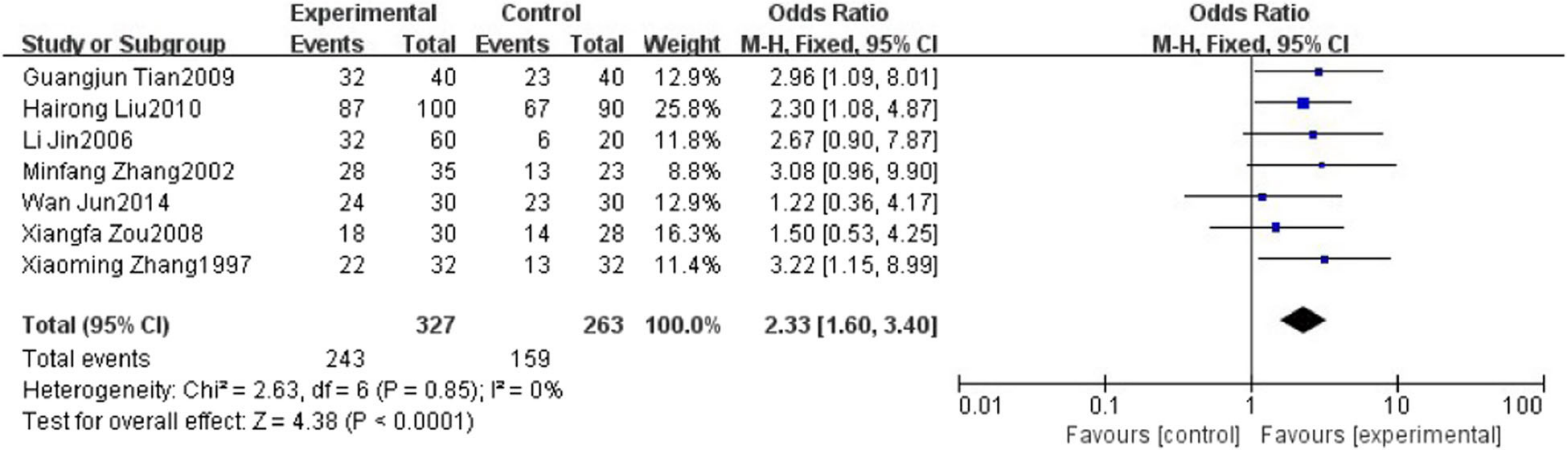
The effect of HXHY therapy on B ultrasonic level in NAFLD patients

Seven studies reported type-B ultrasonic. These studies involved 590 patients including 327 patients in HXHY therapy group and 263 patients in the control group. We found no significant heterogeneity in these studies (퐼I^2^ = 0%, 푃P= 0.85). A fixed effects model analysis showed that HXHY was more beneficial to change type-B ultrasonic level in NAFLD Patients when compared to the conventional treatment group (OR = 2.33; 95% CI: 1.60, 3.40; *P* < 0.0001) (Fig. 3).

### The effect of HuoXueHuaYu therapy on blood lipids in patients with NAFLD

**Fig. 4:**
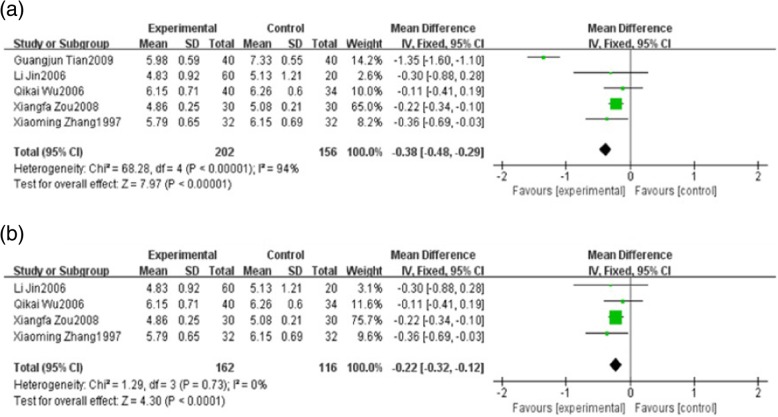
**a** The effect of HXHY on blood lipid total cholesterol in NAFLD patients. **b** Sensitivity analysis was performed by omitting one study

Five studies reported total cholesterol (TC). These studies including 358 patients which 202 patients belonged to the HXHY therapy group and 156 patients belonged to the conventional treatment group. As the results showed, the heterogeneity of TC was high (*I* ^2^ = 94%, *P* < 0.00001) among trials when comparing HXHY therapy with conventional treatment group. The random-effect model analysis showed that patients with NAFLD who received HXHY had significantly lower TC levels than those who received conventional therapy. (MD = − 0.38; 95% CI: − 0.48, − 0.29; *P* < 0.00001) (Fig. 4a). Sensitivity analysis results suggested that the study carried out by Guangjun Tian 2009 made a great contribution to the high heterogeneity. There was no heterogeneity existed when the study was removed (*I*^2^ = 0%, *P* = 0.73) (Fig. 4b). Meanwhile, we found that the duration of the study was 24 weeks which was obviously longer than other studies, indicating that the duration maybe a source of heterogeneity.

**Fig. 5:**
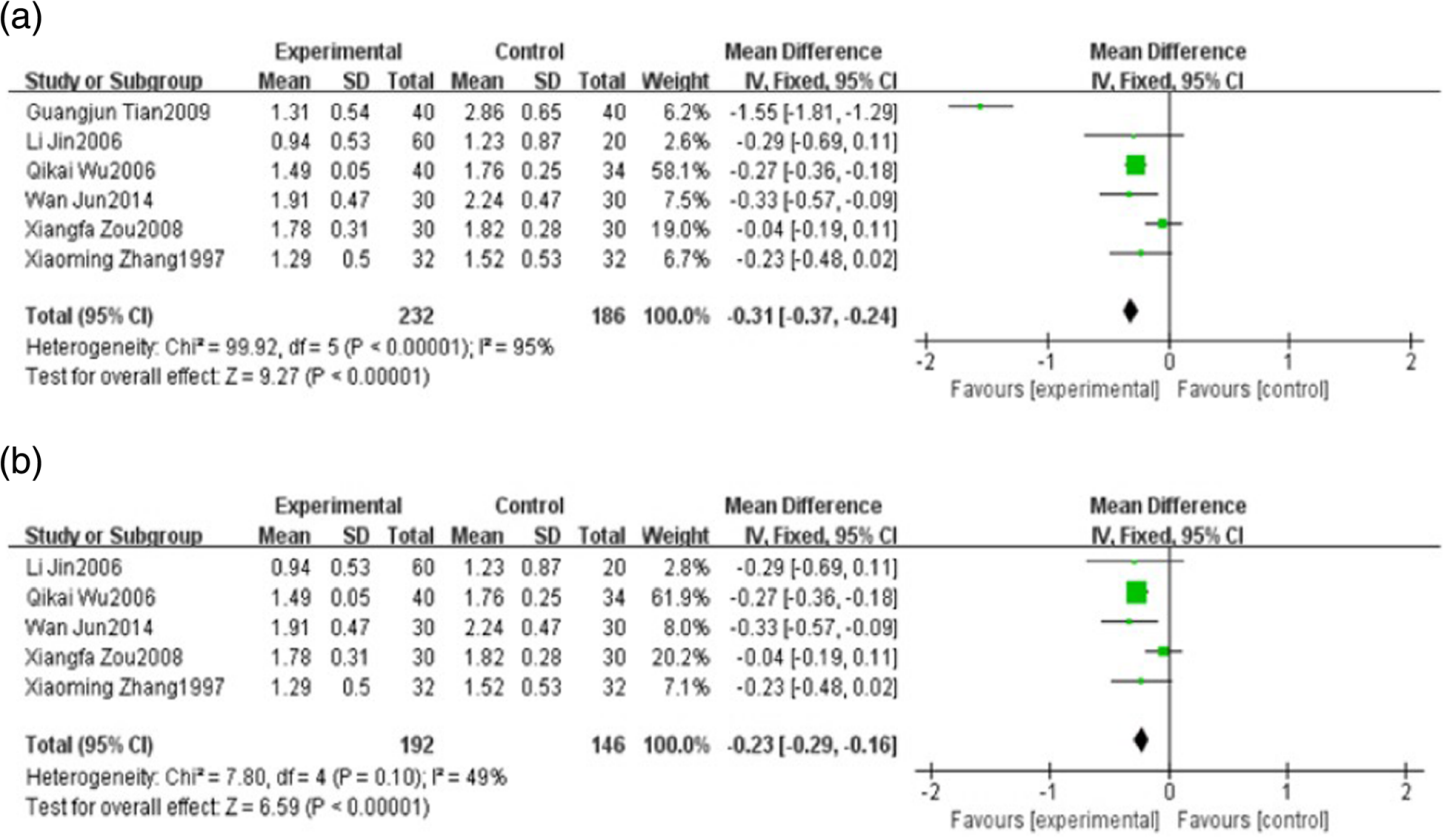
**a** The effect of HXHY on blood lipid triglyceride in NAFLD patients. **b** Sensitivity analysis was performed by omitting one study

Six studies reported triglyceride (TG). These studies including 418 patients which 232 patients belong to the HXHY therapy group and 186 patients belong to the conventional treatment group. We found high significant heterogeneity in TG (*I* ^2^ = 95%, *P* < 0.00001) among trials when comparing HXHY therapy with conventional treatment group. A random-effect model analysis showed that HXHY therapy significantly decrease the level of TG than conventional treatment (MD = − 0.31; 95% CI − 0.37, − 0.24; *P* < 0.00001) (Fig. 5a). Sensitivity analysis results suggested that the study carried out by Guangjun Tian. 2009 made a great contribution to the high heterogeneity. The heterogeneity was much smaller when this study was removed. (*I*^2^ = 49%, *P* = 0.10) (Fig. 5b).

### The effect of HuoxueHuayu therapy on liver function in patients with NAFLD

**Fig. 6:**
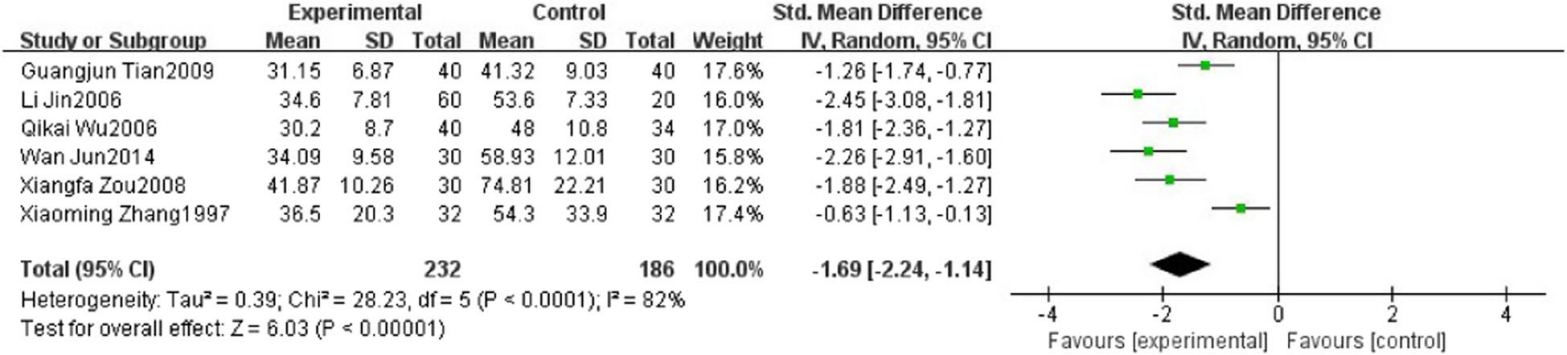
The effect of HXHY on alanine transaminase (ALT) in NAFLD patients

Six studies reported alanine transaminase (ALT). These studies including 418 patients which 232 patients belong to the HXHY therapy group and 186 patients belong to the conventional treatment group. As the results showed, we found high significant heterogeneity in ALT (*I* ^2^ = 82%, *P* < 0.0001) among trials when comparing HXHY therapy with conventional treatment group. A random-effect model analysis showed that HXHY therapy significantly reduce the level of ALT than conventional treatment in the NAFLD patients (MD = − 1.69; 95% CI: − 2.24, − 1.14; *P* < 0.00001) (Fig. 6). Although we conducted sensitivity analysis and subgroup analysis, there was still a high heterogeneity.

**Fig. 7:**
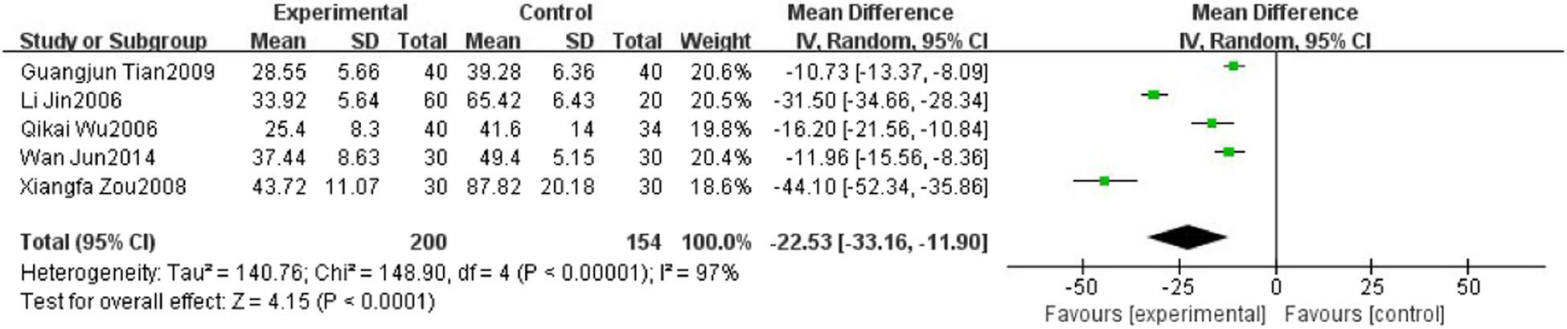
The effect of HXHY on aspartate transaminase (AST) in NAFLD patients

Five studies reported aspartate transaminase (AST). We found high significant heterogeneity in AST (*I*^2^ = 97%, *P* < 0.00001) among trials when comparing HXHY with conventional treatment group. A random-effect model analysis showed that HXHY significantly reduce the level of AST than conventional treatment in the NAFLD patients (MD = − 22.53; 95% CI:− 33.16, − 11.90; *P* < 0.0001) (Fig. 7). Although we conducted sensitivity analysis and subgroup analysis, there was still a high heterogeneity.

### The effect of HuoXueHuaYu therapy on the effective rate in patients with NAFLD

**Fig. 8:**
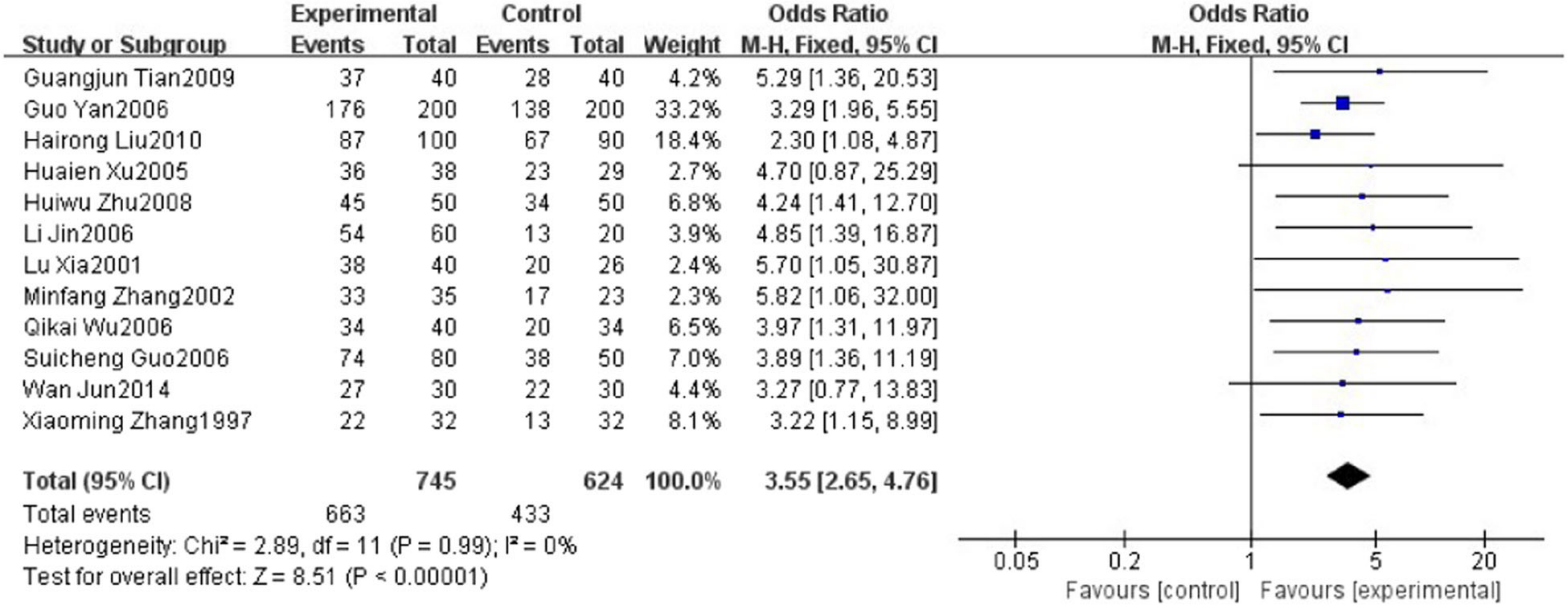
The effect of HXHY therapy on the effective rate in NAFLD patients

**Fig. 9:**
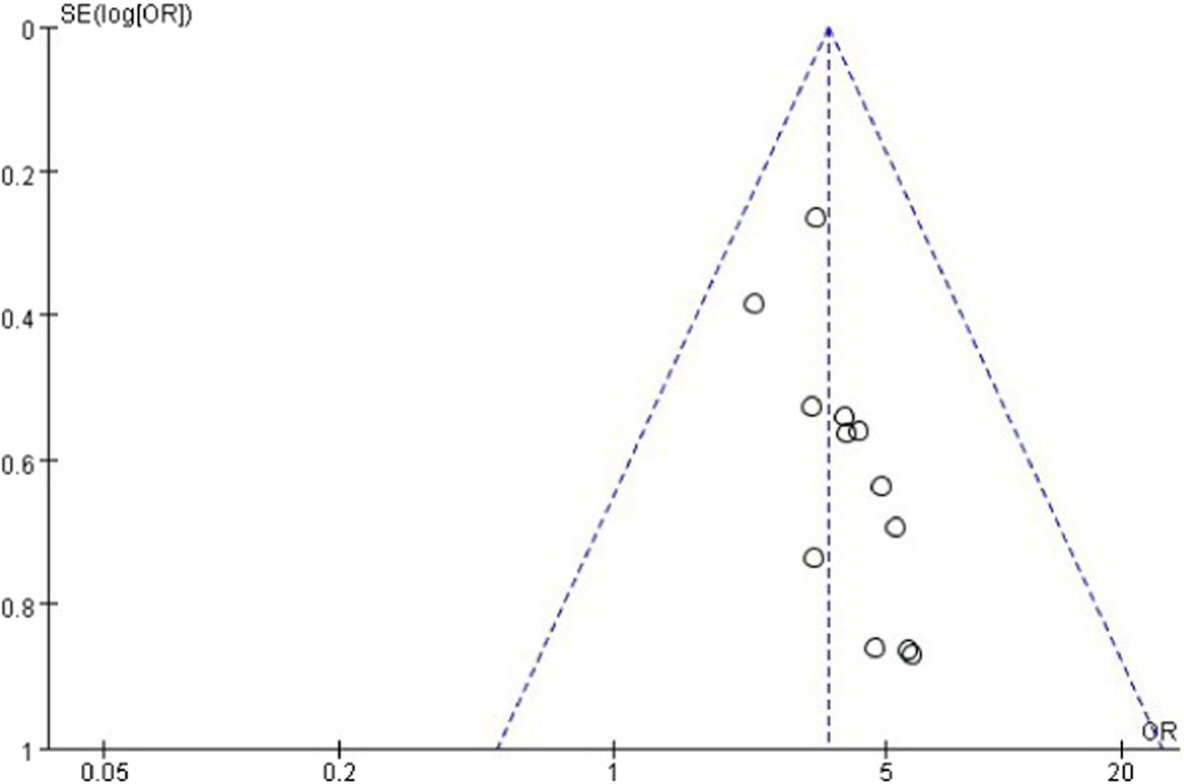
The funnel plot of HXHY therapy on the effective rate in NAFLD patients

Twelve studies reported effective rate of HuoxueHuayu therapy in patients with NAFLD. These studies including 1369 patients which 745 patients belong to the therapy group and 624 patients belong to the conventional treatment group. The effective rate in seven studies [13, 18, 20–23] refers to the proportion of participants with improvement of clinical symptoms and level of type-B ultrasonic of liver. The effective rate in the other five studies [12, 14, 17, 19, 24] refers to the proportion of participants with improvement of clinical symptoms and level of type-B ultrasonic of liver as well as ≥30% reduction in level of liver function and blood lipids. There was no significant heterogeneity with 퐼I^2^ = 0%, *푃P* = 0.99. A fixed effects model analysis showed that HXHY was more beneficial for the effective rate in NAFLD Patients when compared with the conventional treatment group (OR = 3.55; 95% CI:2.65, 4.76; *P* < 0.00001) (Fig. 8). Funnel plot was selected to assess the publication bias for the effective rate, which showed that the distribution is generally almost symmetrical (Fig. 9).

### The safety evaluation of HuoxueHuayu therapy on NAFLD patients

There were no adverse reactions reported in all the 13 articles. Therefore, we may need to assess the safety of HXHY therapy on NAFLD in facilitate further researches.

## Discussion

Based on the meta-analysis of 13 RCTs, it can be documented that HXHY can significantly improve B ultrasound in NAFLD patients when compared with the conventional treatment group. Meanwhile, HXHY therapy also can improve the blood lipid, liver function and the effective rate. Furthermore, there was no obvious adverse reaction reported in treating NAFLD. Our results suggested that HXHY is effective and safe in treating NAFLD.

The mechanism of NAFLD is not fully understood. Recently, researches show that insulin resistance, free radicals and oxidative stress, endoplasmic reticulum stress, and inflammation may involve in the mechanism of NAFLD [25–28]. The general philosophical underlining of Chinese medicine is holistic medicine [29, 30]. TCM has anti-inflammatory effect and high safety in treating chronic liver diseases [31, 32]. Previous studies suggested that HXHY therapy can protect hepatic cells, improve liver function and control the development of hepatic fibrosis. For example, salvianolic acid B extracted from Radix Salvia miltiorrhiza were demonstrated to attenuate liver damage, hepatic steatosis, and reduce the levels of pro-inflammatory cytokines [33]. Hawthorn leaf flavonoids significantly lowered liver/body weight ratio, improved serum parameters and liver dysfunction and alleviated hepatic lipid accumulation [34].

Our study had some limitations. First, the quality of the included trials was generally not high. None of the studies provided the methods of blinding and allocation concealment [35]. Clinical trials should be reported in accordance with the Consolidated Standards of Reporting Trials (CONSORT) standards [36, 37]. Second, the treatment duration of most studies was short. Because NAFLD is chronic disease, longer duration should be taken to assess the safety and effectiveness of HXHY in the treatment of NAFLD. The sensitivity analysis suggested that treatment duration may be the main source of heterogeneity. Third, the sample size of some of the included studies is small. It is necessary to demonstrate whether the effects of HXHY will not be changed in future large-scale trials. Fourth, a wide variety of drugs were used in the control group across the studies, which may be another important source of the heterogeneity. Despite the limitations of this study, to the best of our knowledge, this is the first study to evaluate HXHY therapy for NAFLD.

## Conclusions

In conclusion, this study indicates that HXHY therapy is more effective compared with conventional treatment for patients with NAFLD, suggesting that HXHY may be a new option for treating NAFLD. Due to the pool quality of the included studies, it is necessary to validate the conclusions by more rigorously designed, multi-centered RCTs with high quality.

## Data Availability

All data generated or analysed during this study are included in this published article.

## Abbreviations

ALT: Alanine transaminase
AST: Aspartate transaminase
CI: Confidence interval
CNKI: Chinese journal full-text database
HXHY: HuoXueHuaYu
MD: Mean difference
NAFLD: Nonalcoholic fatty liver disease
NASH: Nonalcoholic steatohepatitis
OR: Odds ratio
RCTs: Randomized controlled trials
TC: Total cholesterol
TCM: Traditional Chinese medicine
TG: Triglyceride
